# Supplementary material for: An integrated genomic analysis of anaplastic meningioma identifies prognostic molecular signatures
Source: Sci Rep. 2018 Sep 10;8:13537. doi: 10.1038/s41598-018-31659-0 (PMC6131140; doi:10.1038/s41598-018-31659-0)
Supplement: Supplementary file 1 [file 41598_2018_31659_MOESM1_ESM.pdf]

An integrated genomic analysis of anaplastic meningioma identifies prognostic molecular signatures

Grace Collord[1,2,22], Patrick Tarpey[1,22], Natalja Kurbatova[3], Inigo Martincorena[1], Sebastian Moran[4], Manuel Castro[4], Tibor Nagy[1], Graham Bignell[1], Francesco Maura[1,5,6], Matthew D. Young[1], Jorge Berna[7], Jose M. Tubio[7], Chris E. McMurran[8], Adam M.H. Young[8], Mathijs Sanders[1,20], Imran Noorani[1,8], Stephen J Price[8], Colin Watts[8], Elke Leipnitz[9], Matthias Kirsch[9], Gabriele Schackert[9], Danita Pearson[10], Abel Devadass[10], Zvi Ram[16], V. Peter Collins[10], Kieren Allinson[10], Michael D. Jenkinson[11,19], Rasheed Zakaria[11,12], Khaja Syed[11,12], C. Oliver Hanemann[13], Jemma Dunn[13], Michael W. McDermott[14], Ramez W Kirollos[8], George S. Vassiliou[1,15,], Manel Esteller[4,17,18], Sam Behjati[1,2], Alvis Brazma[3], Thomas Santarius[8*], Ultan McDermott[1,21*]

[1] Wellcome Sanger Institute, Wellcome Genome Campus, Hinxton, CB10 1SA, UK
[2] Department of Paediatrics, University of Cambridge, Cambridge Biomedical Campus, CB2 0QQ, UK
[3] European Molecular Biology Laboratory, European Bioinformatics Institute, EMBL-EBI, Wellcome Trust Genome Campus, Hinxton, CB10 1SD, UK
[4] Cancer Epigenetics and Biology Program (PEBC), Bellvitge Biomedical Research Institute (IDIBELL), L'Hospitalet de Llobregat, Barcelona, Catalonia, Spain
[5] Department of Oncology and Hemato-Oncology, University of Milan, Milan, Italy
[6] Department of Hematology, Fondazione IRCCS Istituto Nazionale dei Tumori, Milan, Italy
[7] Phylogenomics Lab, Edificio Torre CACTI, Campus Universitario, Universidad de Vigo, 36310 Vigo, Spain
[8] Department of Neurosurgery, Department of Clinical Neuroscience, Cambridge University Hospitals NHS Foundation Trust, Cambridge, CB2 0QQ, UK
[9] Klinik und Poliklink für Neurochirurgie, "Carl Gustav Carus" Universitätsklinikum, Technische Universität Dresden, Fetscherstrasse 74, 01307 Dresden, Germany
[10] Department of Pathology, Cambridge University Hospital, CB2 0QQ, Cambridge, UK
[11] Department of Neurosurgery, The Walton Centre, Liverpool, L9 7LJ, UK
[12] Institute of Integrative Biology, University of Liverpool, Liverpool, L9 7LJ, UK
[13] Institute of Translational and Stratified Medicine, Plymouth University Peninsula Schools of Medicine and Dentistry, Plymouth University, Plymouth, Devon PL4 8AA, UK
[14] Department of Neurosurgery, UCSF Medical Center, San Francisco, CA 94143-0112, USA
[15] Department of Haematology, Cambridge University Hospitals NHS Trust, Cambridge, CB2 0QQ, UK
[16] Department of Neurosurgery, Tel-Aviv Medical Center, Tel-Aviv, Israel
[17] Physiological Sciences Department, School of Medicine and Health Sciences, University of Barcelona (UB), Catalonia, Spain
[18] Institució Catalana de Recerca i Estudis Avançats (ICREA), Barcelona, Catalonia, Spain
[19] Institute of Translational Medicine, University of Liverpool, Liverpool, L9 7LJ, UK
[20] Erasmus University Medical Center, Department of Hematology, Rotterdam, The Netherlands
[21] Current address: AstraZeneca, CRUK Cambridge Institute, Robinson Way, Cambridge, UK CB2 0RE
[22] These authors contributed equally to this work.

[*] Corresponding authors

Supplementary Figure S1

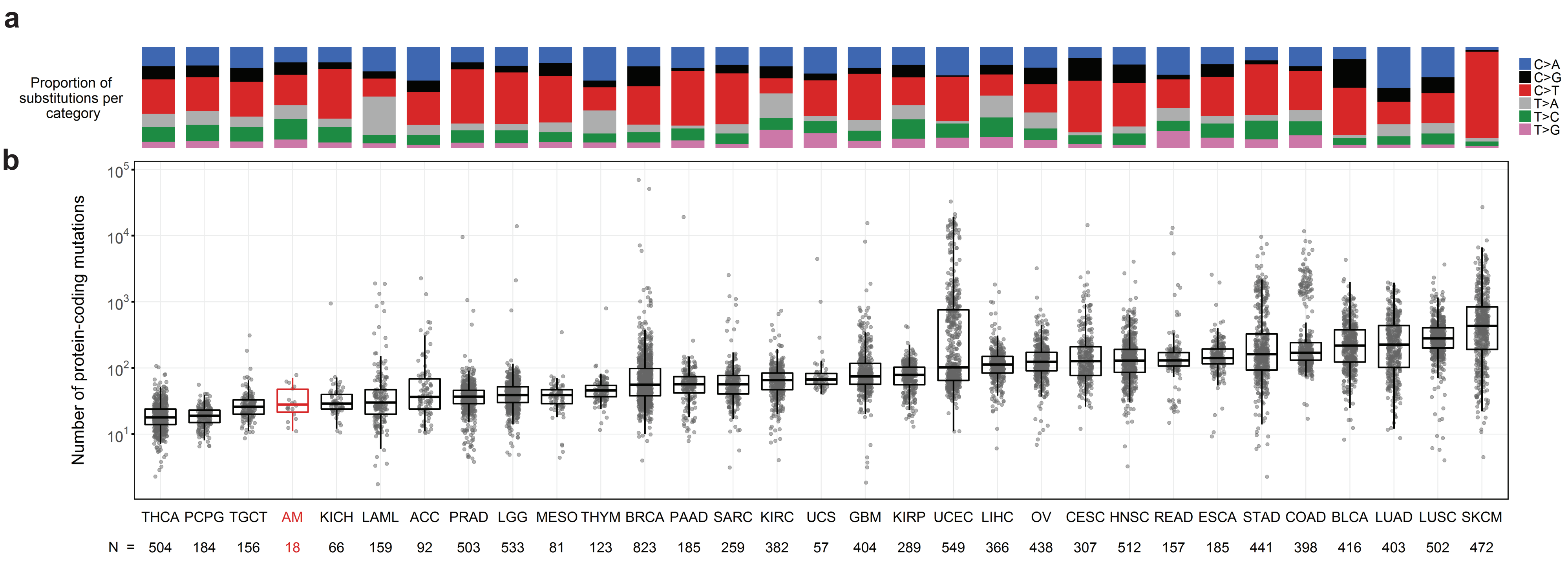

Supplementary Figure S1 | Anaplastic meningiomas have a modest point mutation burden relative to other cancers. **(a)** Proportion of substitutions classified according to pyrimidine base change. **(b)** Protein-coding point mutation burden of anaplastic meningioma relative to similar estimates obtained from TCGA datasets for 30 other cancer types. The horizontal axis indicates tumor type and number of samples with anaplastic meningioma indicated in red. The vertical axis shows the number of protein-coding mutations (Log_{10}). Box plot hinges demarcate the 25th to 75th centiles with median indicated by the middle horizonal line and whiskers extending to 1.5 times the interquartile range. Individual points are tumor samples. AM, anaplastic meningioma; TCGA abbreviations are detailed in Supplementary Table S2.

Supplementary Figure S2

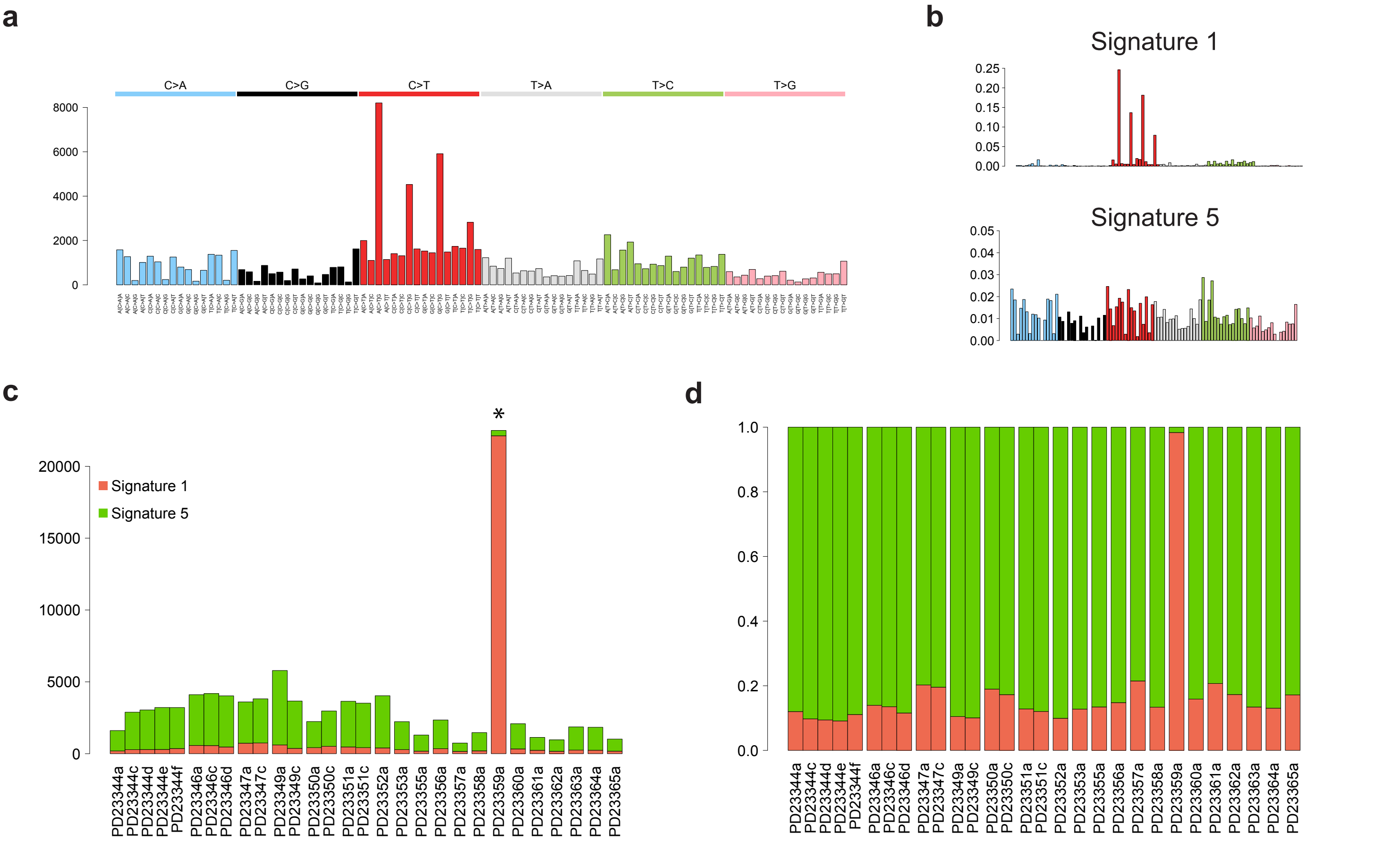

Supplementary Figure S2 | Mutational signature analysis of anaplastic meningioma. (a) Barplot showing the frequency of substitutions classed by trinucleotide context (n = 29 anaplastic meningioma genes). **(b)** Substitution pattern of extracted signatures 1 and 5. **(c-d)** The absolute number (c) and relative proportion (d) of substitutions contributing to each signature per tumor sample (*hypermutator tumor PD23359a).

Supplementary Figure S3

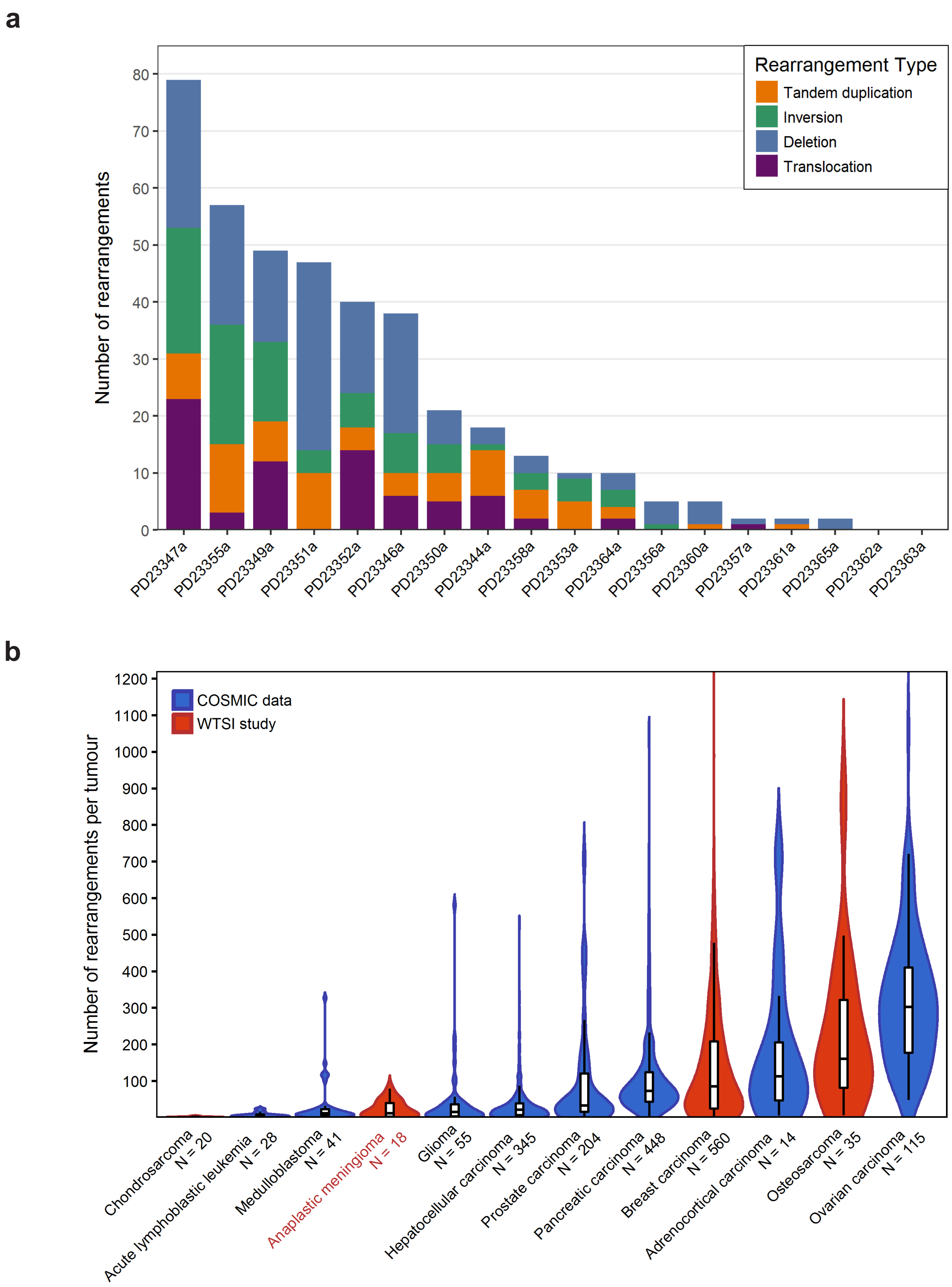

Supplementary Figure S3 | Rearrangement burden of anaplastic meningioma. (a) Rearrangement subtypes in 18 primary anaplastic meningioma genomes classified into four broad categories of structural variant. The horizontal axis indicates sample number and the vertical axis shows the total number of rearrangements. **(b)** Comparison of overall rearrangement burden in anaplastic meningioma with 11 other tumor types colour coded according to source of data and analysis. Horizontal axis indicates cancer type and number of samples per cohort. Vertical axis shows rearrangent number per sample. Box plot hinges demarcate the 25th to 75th centile with median indicated by the middle horizonal line and whiskers extending to 1.5 times the interquartile range. Underlying violin plots show the full range and distribution of rearrangement burden across each tumor cohort. WTSI, Wellcome Trust Sanger Institute.

Supplementary Figure S4

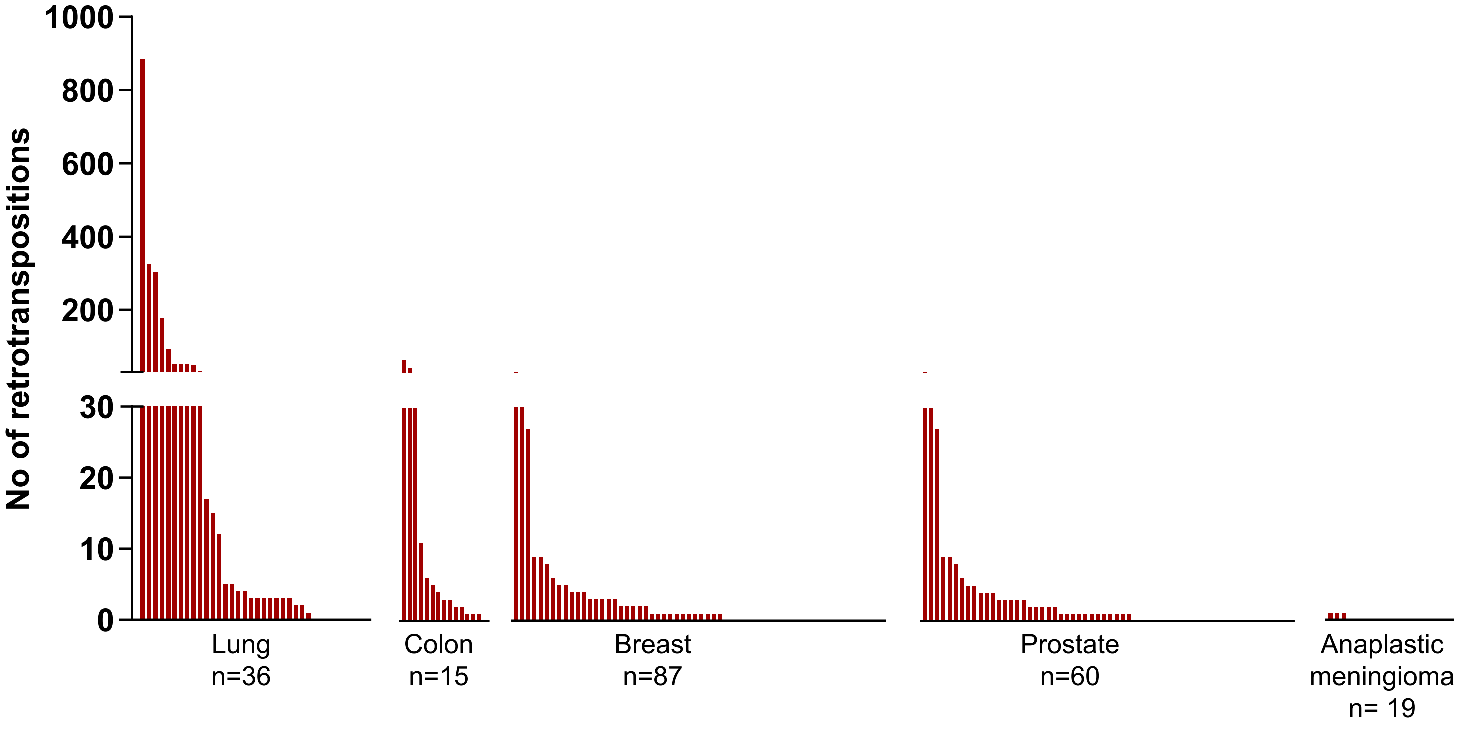

Supplementary Figure S4 | Somatic L1 retrotranspositon burden in anaplastic meningioma relative to 4 other cancer types. Distribution of L1 retrotranspositions detected in 19 primary anaplastic meningioma genomes and 198 previously published genomes encompassing four tumor types. X-axis indicates cancer type and number of samples in the cohort. Y-axis indicates total number of retrotranspositions detected per genome.

a

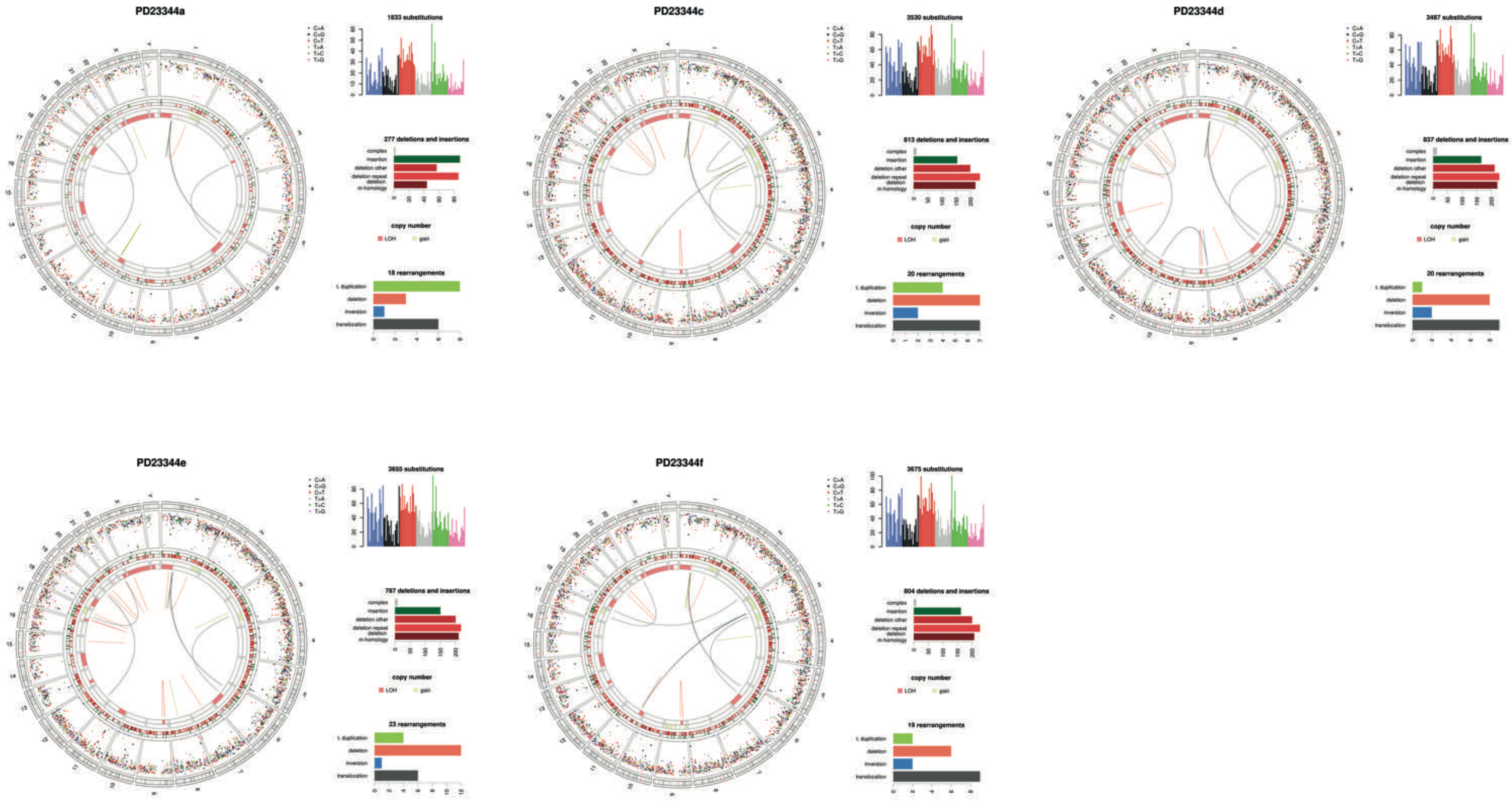

Supplementary Figure S5 | Circos plots of 31 anaplastic meningioma genomes. (a - f) Paired primary and recurrent tumour(s) from 6 individuals. (g) Three relapse specimens from a patient with no available primary tumour. **(h - s)** Primary tumours without paired recurrences. All substitution mutations are represented by their intermutation distance per chromosome and are color coded according to which base substitution (C>A, C>G, C>T, T>A, T>C, T>G). The six concentric circles in each plot, from outermost inwards, represent the human chromosomes, substitutions, insertions, deletions and copy number for each allele. The three bar plots to the right of each circos depict, in descending order, substitution mutation trinucleotide context and burden of small indels and rearrangements.

b

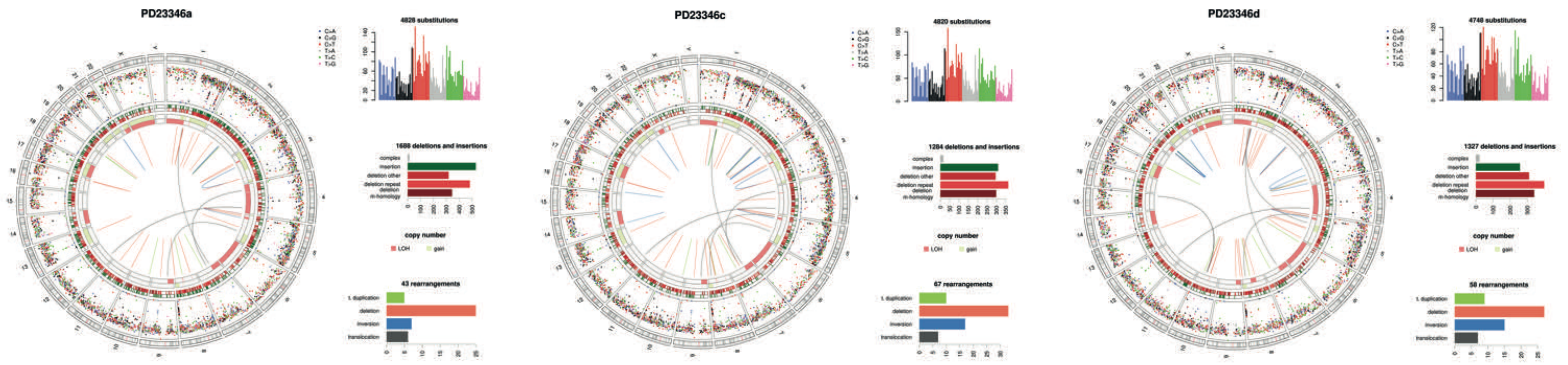
PD23346a
4828 substitutions
1688 deletions and insertions
copy number
LOH
gain
43 rearrangements
PD23346c
4820 substitutions
1284 deletions and insertions
copy number
LOH
gain
67 rearrangements
PD23346d
4748 substitutions
1327 deletions and insertions
copy number
LOH
gain
58 rearrangements

c

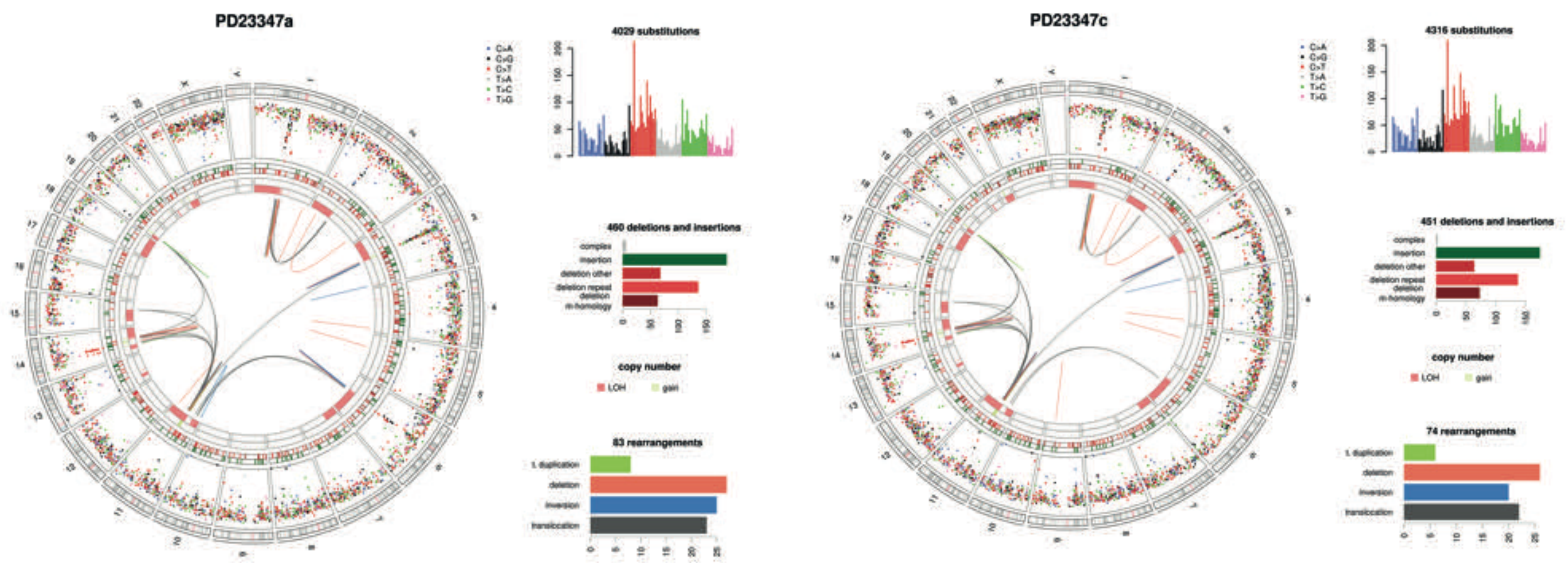
PD23347a
4029 substitutions
460 deletions and insertions
copy number
LOH
gain
83 rearrangements
PD23347c
4316 substitutions
451 deletions and insertions
copy number
LOH
gain
74 rearrangements

d

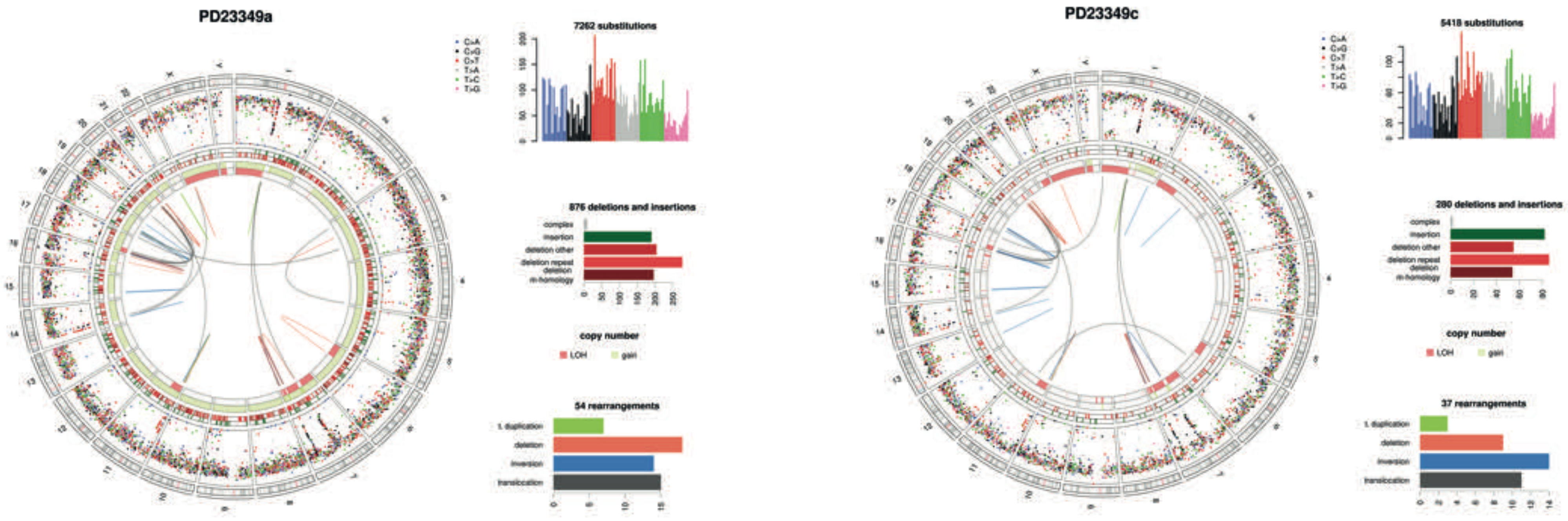

PD23349a
C>A
C>G
C>T
T>A
T>C
T>G
7262 substitutions
876 deletions and insertions
complex
insertion
deletion other
deletion repeat
deletion
mh-homology
copy number
LOH
gain
54 rearrangements
t. duplication
deletion
inversion
translocation
PD23349c
5418 substitutions
280 deletions and insertions
37 rearrangements

e

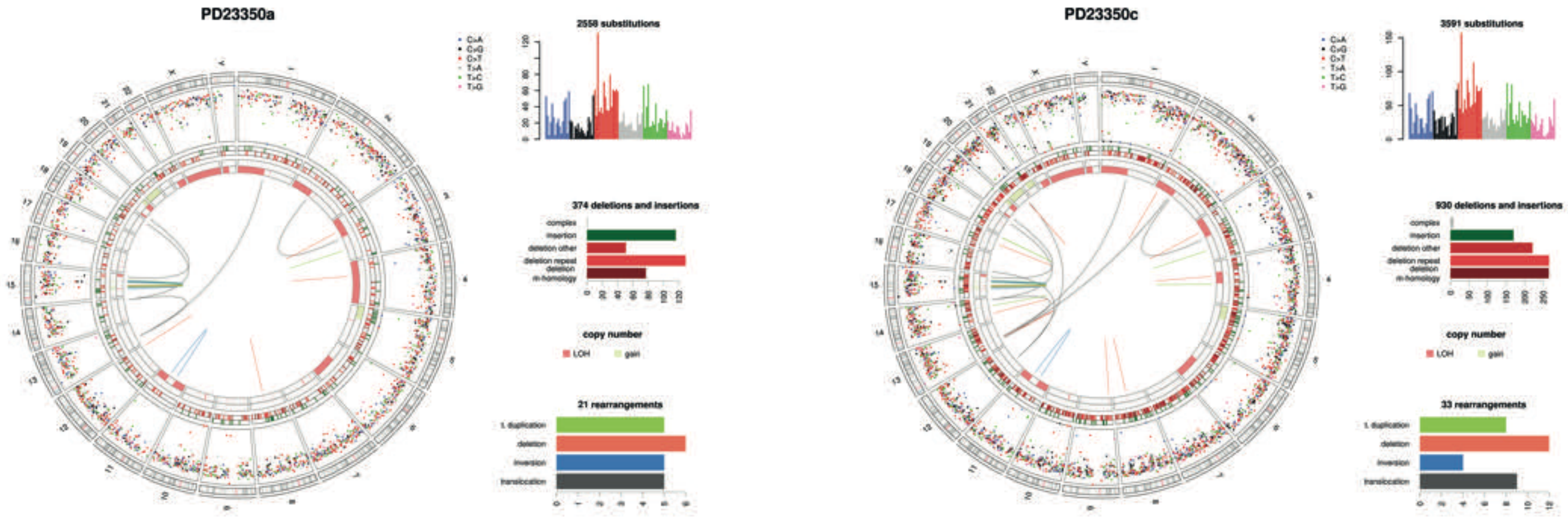

PD23350a
C>A
C>G
C>T
T>A
T>C
T>G
2558 substitutions
374 deletions and insertions
complex
insertion
deletion other
deletion repeat
deletion
mh-homology
copy number
LOH
gain
21 rearrangements
t. duplication
deletion
inversion
translocation
PD23350c
3591 substitutions
930 deletions and insertions
33 rearrangements

f

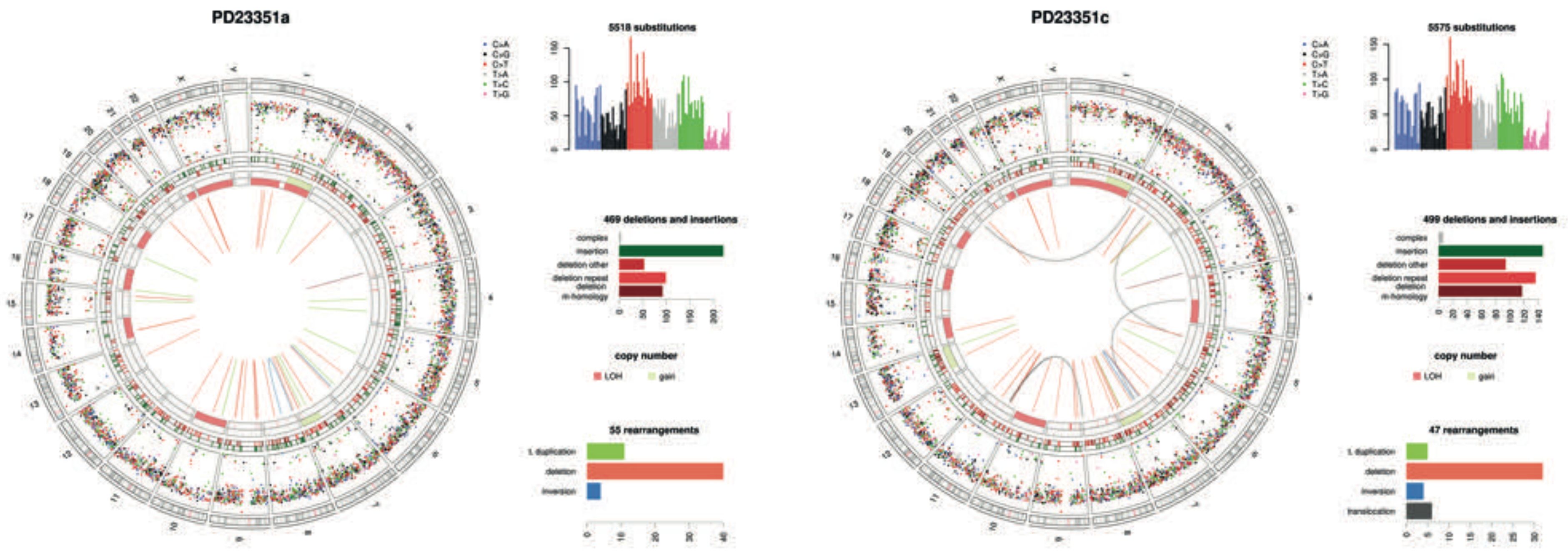
PD23351a
C>A
C>G
C>T
T>A
T>C
T>G
5518 substitutions
469 deletions and insertions
complex
insertion
deletion other
deletion repeat
deletion
mi-homology
copy number
LOH
gain
55 rearrangements
t. duplication
deletion
inversion
PD23351c
5575 substitutions
499 deletions and insertions
47 rearrangements
translocation

g

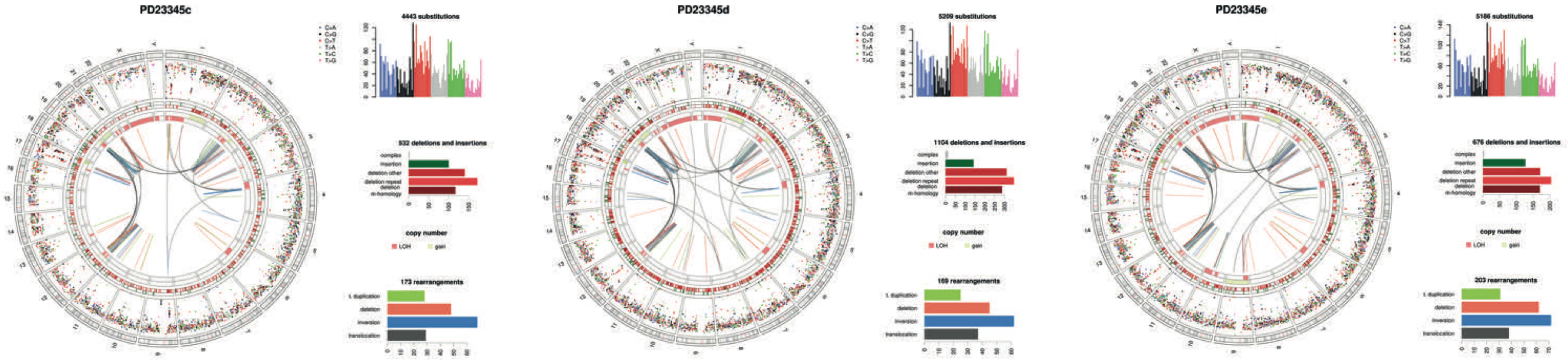
PD23345c
4443 substitutions
532 deletions and insertions
copy number
LOH
gain
173 rearrangements
t. duplication
deletion
inversion
translocation
PD23345d
5209 substitutions
1104 deletions and insertions
169 rearrangements
PD23345e
5186 substitutions
676 deletions and insertions
203 rearrangements

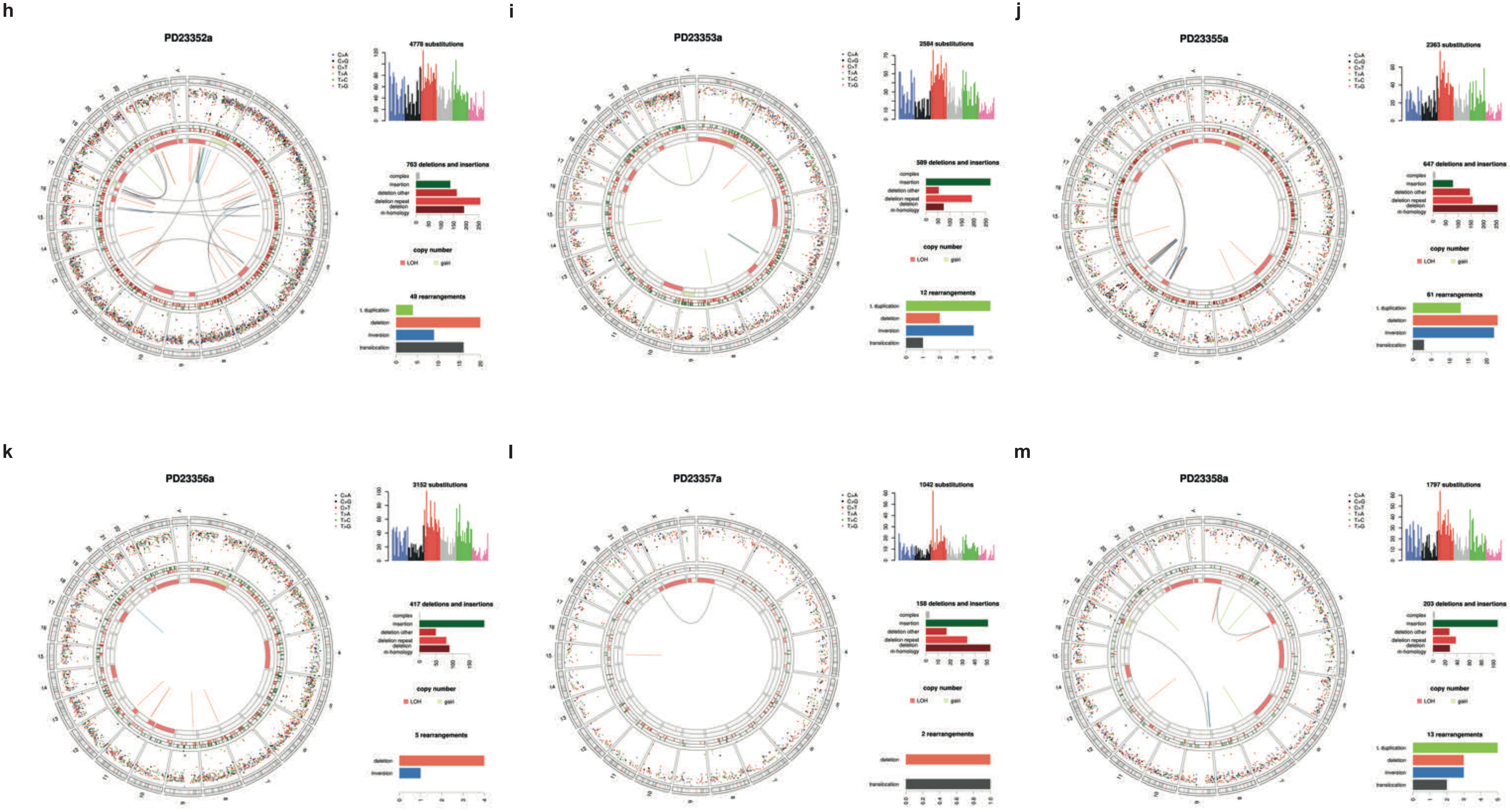
h
PD23352a
4778 substitutions
763 deletions and insertions
copy number
LOH
gain
49 rearrangements
i
PD23353a
2584 substitutions
589 deletions and insertions
12 rearrangements
j
PD23355a
2363 substitutions
647 deletions and insertions
61 rearrangements
k
PD23356a
3152 substitutions
417 deletions and insertions
5 rearrangements
l
PD23357a
1042 substitutions
158 deletions and insertions
2 rearrangements
m
PD23358a
1797 substitutions
203 deletions and insertions
13 rearrangements

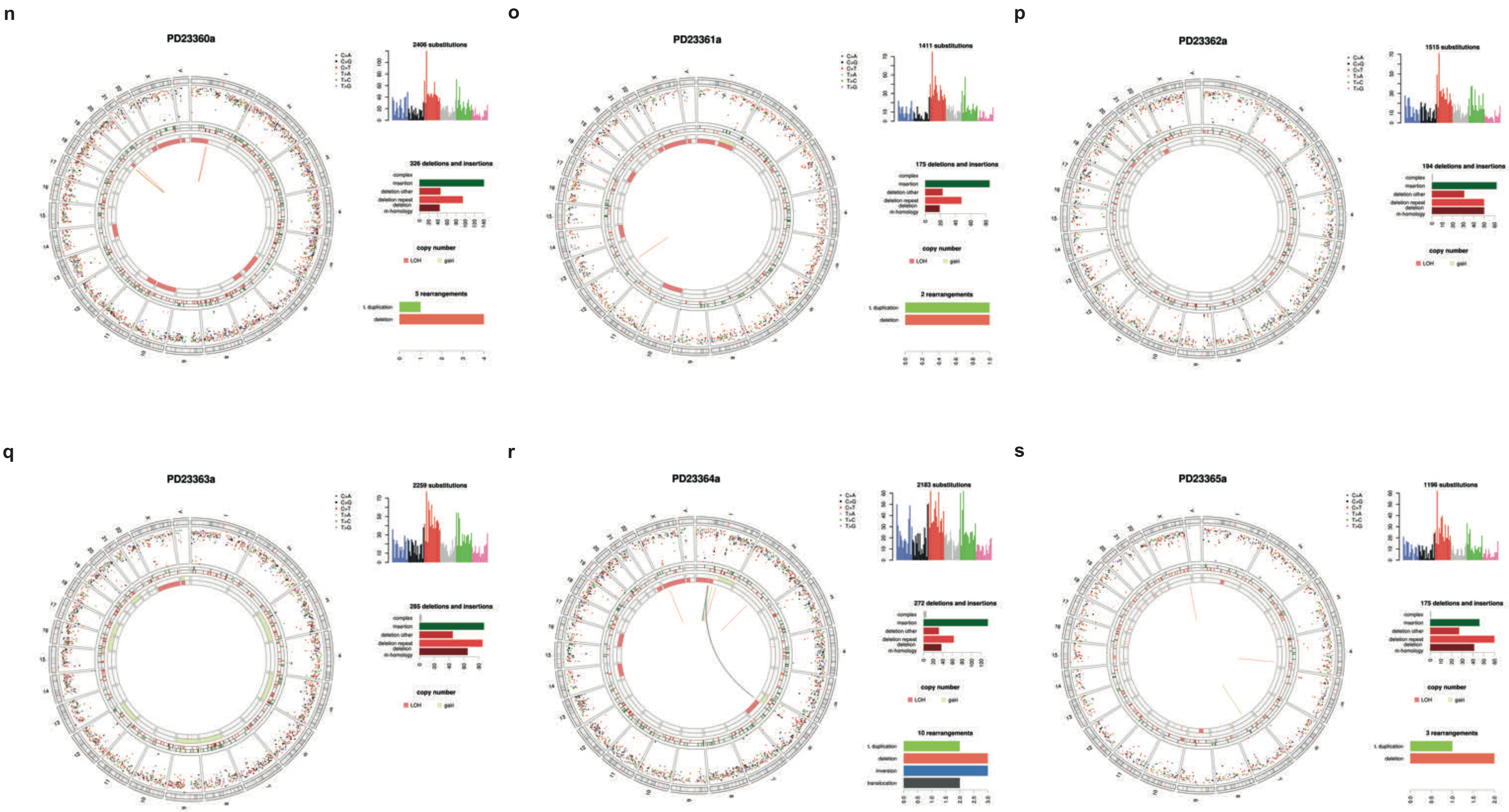
n
PD23360a
2406 substitutions
326 deletions and insertions
copy number
LOH
gain
5 rearrangements
t. duplication
deletion
o
PD23361a
1411 substitutions
175 deletions and insertions
copy number
LOH
gain
2 rearrangements
t. duplication
deletion
p
PD23362a
1515 substitutions
194 deletions and insertions
copy number
LOH
gain
q
PD23363a
2259 substitutions
285 deletions and insertions
copy number
LOH
gain
r
PD23364a
2183 substitutions
272 deletions and insertions
copy number
LOH
gain
10 rearrangements
t. duplication
deletion
inversion
translocation
s
PD23365a
1196 substitutions
175 deletions and insertions
copy number
LOH
gain
3 rearrangements
t. duplication
deletion

Supplementary Figure S6

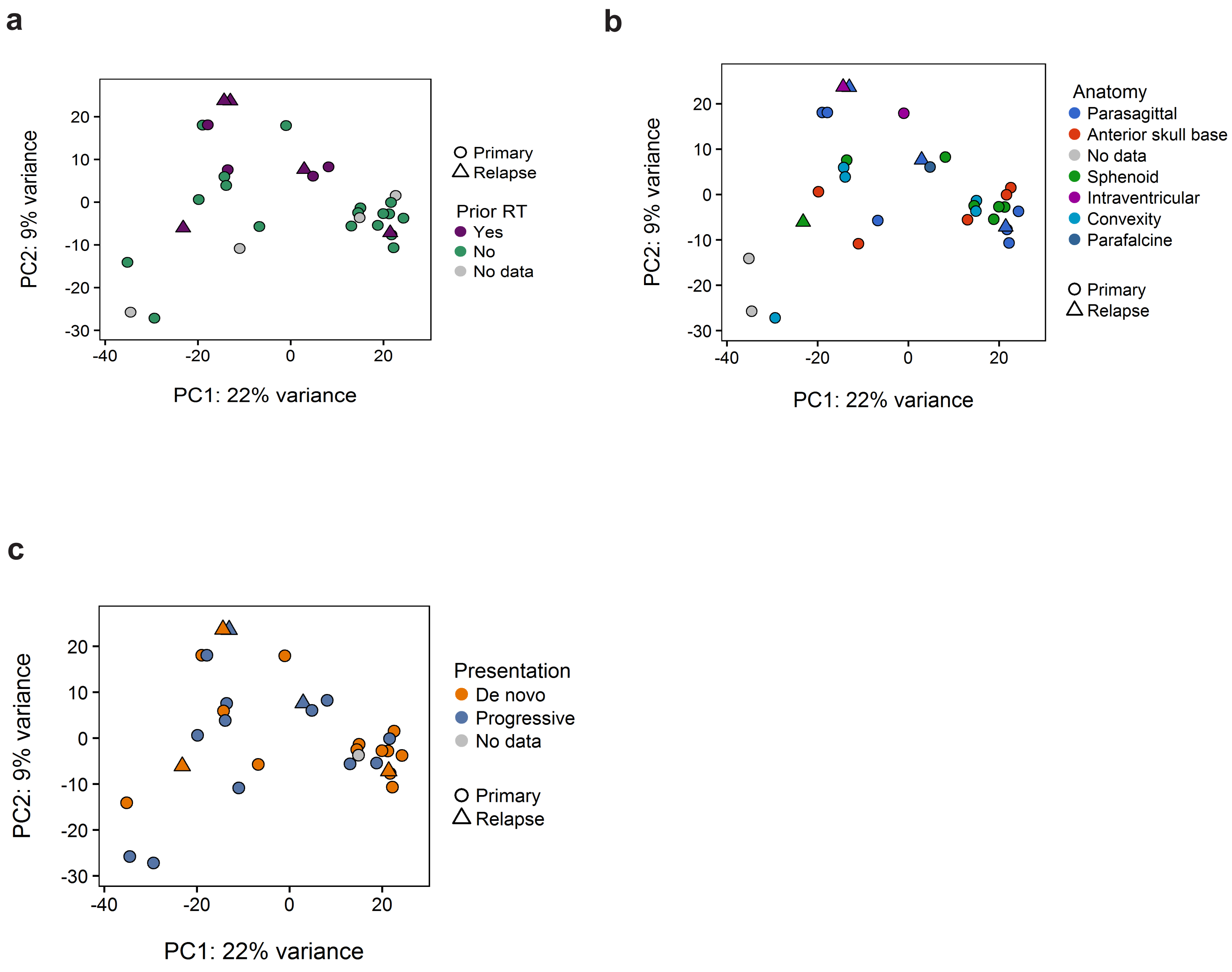

Suplementary Figure S6 | Principal component analysis of anaplastic meningioma transcriptomic data does not reveal molecular subgroups defined by clinical features. Samples are indicated with circles (primary tumor) or triangles (recurrent tumour) and coloured according to **(a)** prior radiotherapy treatment, **(b)** anatomical location and **(c)** whether *de novo* presentation or progression from prior grade 1 or grade 2 meningioma. RT, radiotherapy; PC, principal component.

Supplementary Figure S7

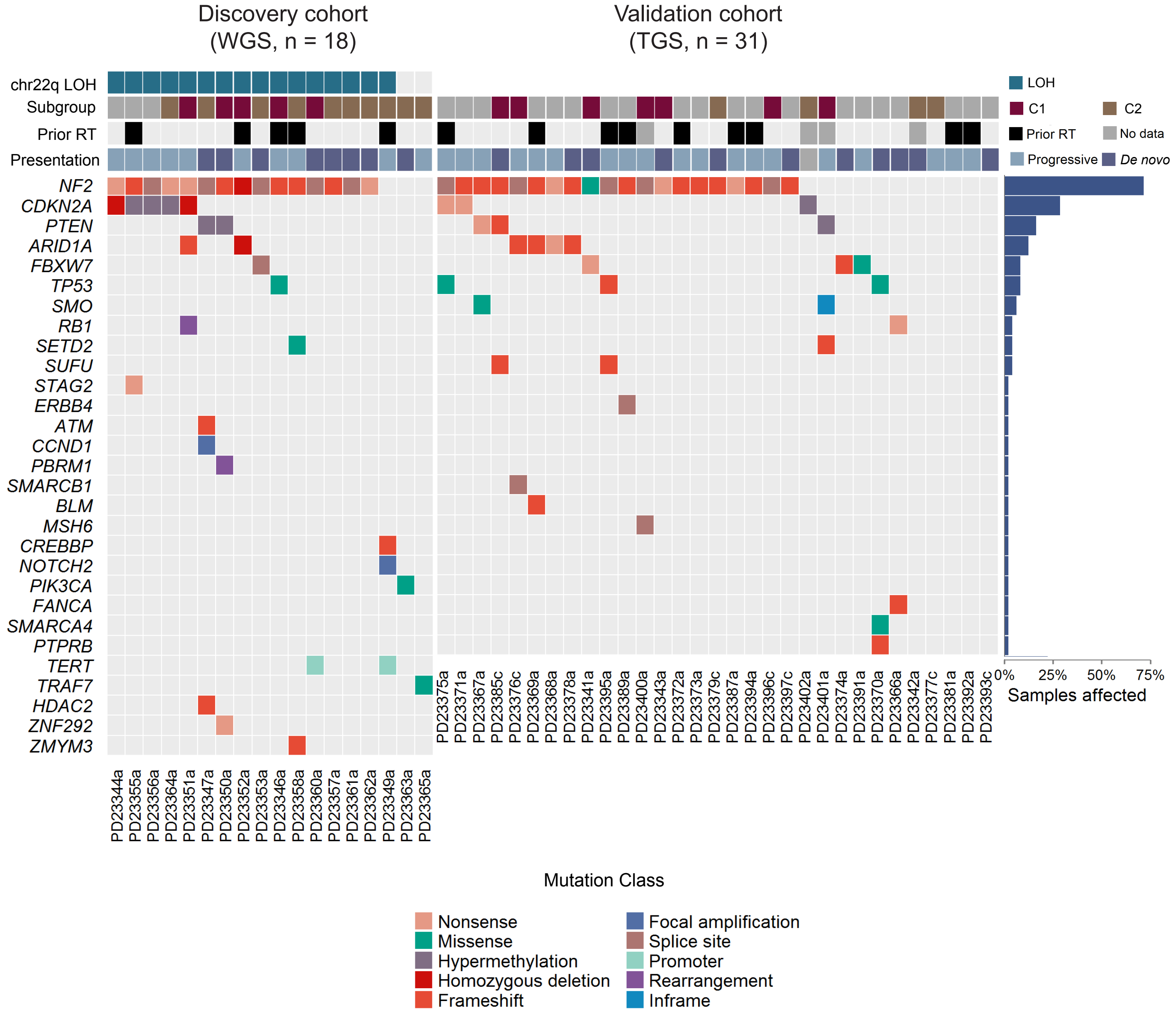

Supplementary Figure S7 | All putative driver variants detected in 49 primary anaplastic meningiomas. Somatic mutation and promoter methylation data is shown for 49 primary tumors, 18 interrogated with whole genome sequencing (discovery cohort), and 31 by targeted sequencing (TGS) of 366 cancer genes. All putative driver mutations are shown, including all variants in genes mutated in a single tumor. The upper panel indicates chromosome 22q LOH status (where reliably detectable), C1/C2 subgroup classification according to gene expression profile, previous radiotherapy treatment, and clinical presentation (whether *de novo* or progression from a lower grade meningioma). The barchart to the right indicates the overall frequency of mutations in each gene across all 49 primary tumors. WGS, whole genome sequencing; TGS, targeted gene sequencing; LOH, loss of heterozygosity; RT, radiotherapy.

Supplementary Figure 8

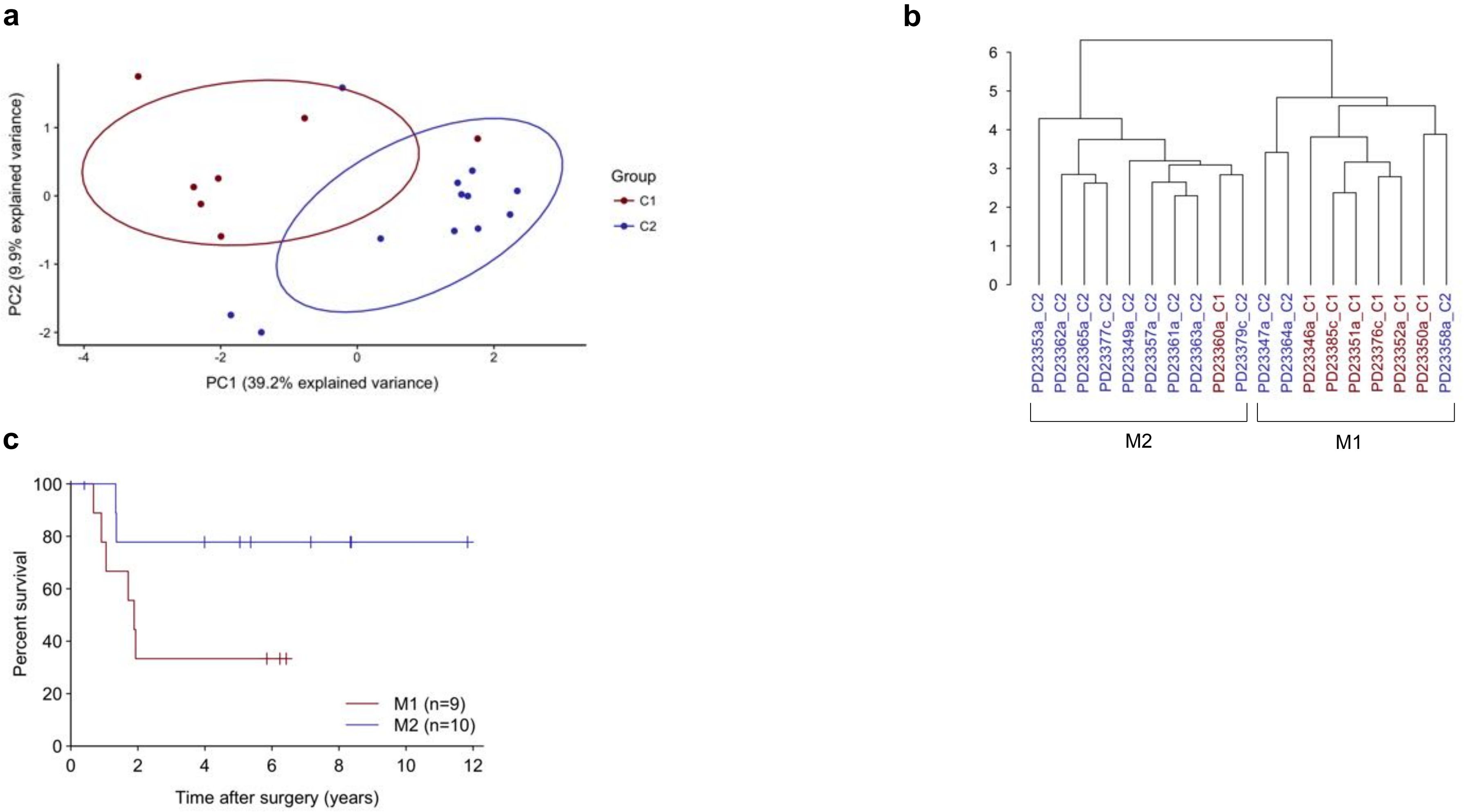

Supplementary Figure 8 | Segregation of 19 anaplastic meningioma samples by methylation profile.
a) Principal component analysis of methylation beta values. Samples are colored according to subgroup derived from gene expression profile. For each group a normal data ellipse is plotted; the size of each ellipse is defined as a normal probability equal to 0.68. **b)** Unsupervised hierarchical clustering using methylation beta values with transcriptomic subgroup indicated after the underscore following each sample name. The methylation-based sample groups are defined by the first bifurcation of the dendrogram as M1 and M2. **c)** Kaplan-Meier curves showing overall survival for 19 anaplastic meningioma patients in M1 and M2 subgroups defined by unsupervised hierarchical clustering of methylation beta values in (b). Dashes indicate timepoints at which subjects were censored at time of last follow-up. PC, principal component.

Supplementary Figure S9

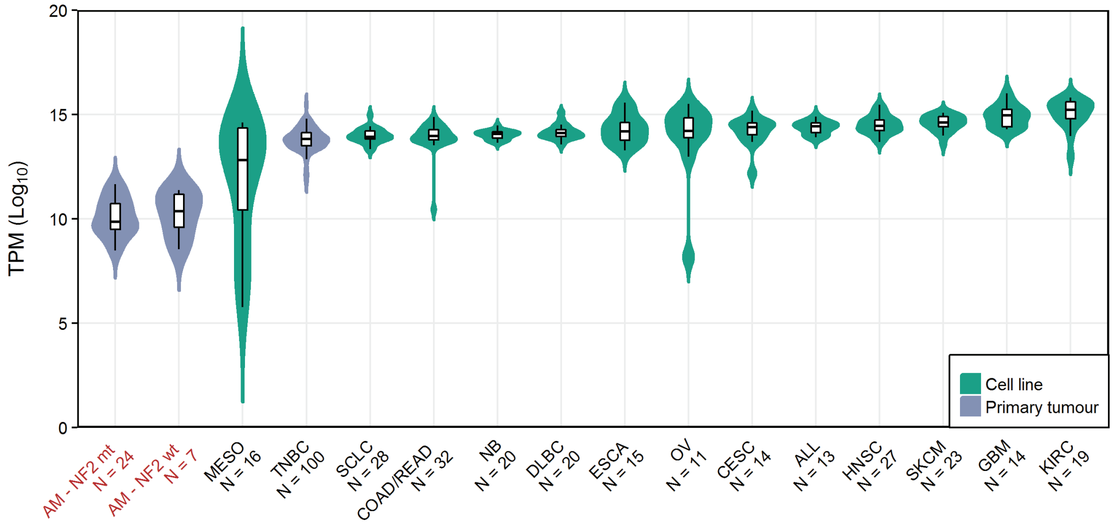

Supplementary Figure S9 | Anaplastic meningiomas demonstrate low NF2 expression regardless of *NF2* mutation status. Box plots of NF2 expression across 31 anaplastic meningomas, 100 primary breast tumors, and 252 cancer cell lines from 13 tumor types. Upper and lower box hinges correspond to first and third quartiles, horizontal line and whiskers indicate the median and 1.5-fold the interquarntile range, respectively. Underlying violin plots show data distribution and are colour-coded according to specimen source (green, cell line; blue, pimary tumor). X-axis indicates tumor type and number of samples in cohort. Y-axis shows NF2 Log_{10} TPM value prevalence. TPM, transcripts per kilobase million; AM, anaplastic meningioma; TNBC, triple negative breast carcinoma; wt, wild-type; mt, mutated.

Supplementary Figure S10

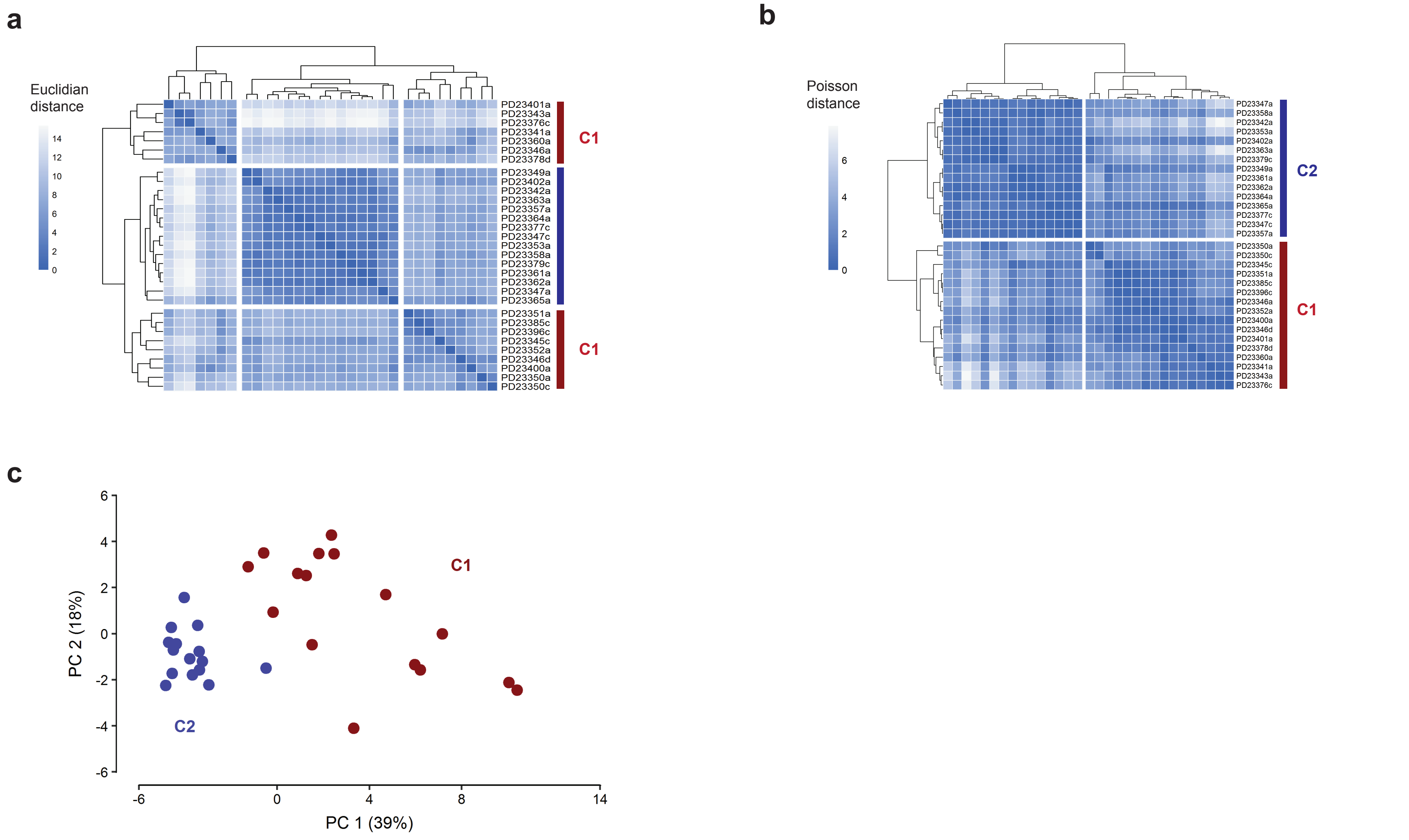

Supplementary Figure S10 | Unsupervised hierarchical clustering and PCA of anaplastic meningiomas using expression level of 6 transcripts recapitulates C1 and C2 subgroups. (a) Unsupervised hierarchical clustering based on Euclidian distance metric. (b) Unsupervised hierarchical clustering based on Poisson distance metric. (c) Principal component analysis; C1 and C2 denote meningioma subgroups identified by unsupervised hierarchical clustering approaches. PC, principal component.

Supplementary Figure S11

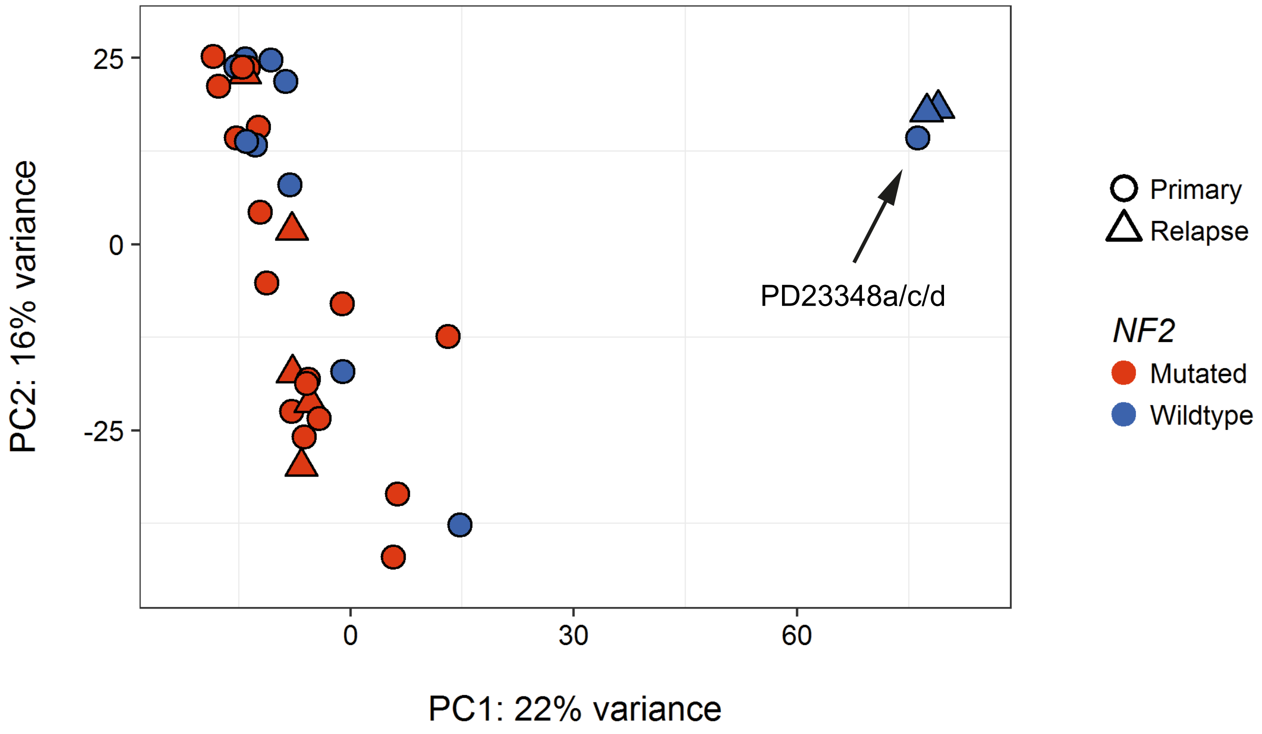

Supplementary Figure S11 | Gene expression profiling aids identifiation of 3 misdiagnosed tumors. Plot of the first two principal components representing 38% of the total variance in the transcriptome data set from 34 tumors included in the initial cohort. Three tumors from individual PD23348 (primary and two subsequent recurrences) clustered separately and were subsequently found to harbour the canonical *NAB2-STAT6* gene fusion that is pathognomonic for meningeal hemangiopericytomas. PC, principal component.

Supplementary S12

a

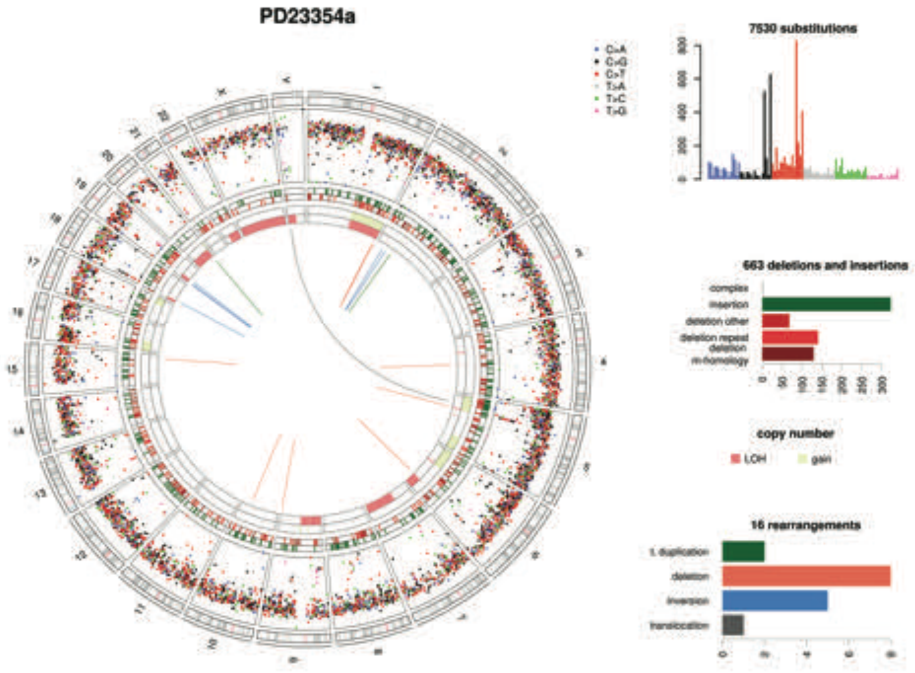

b

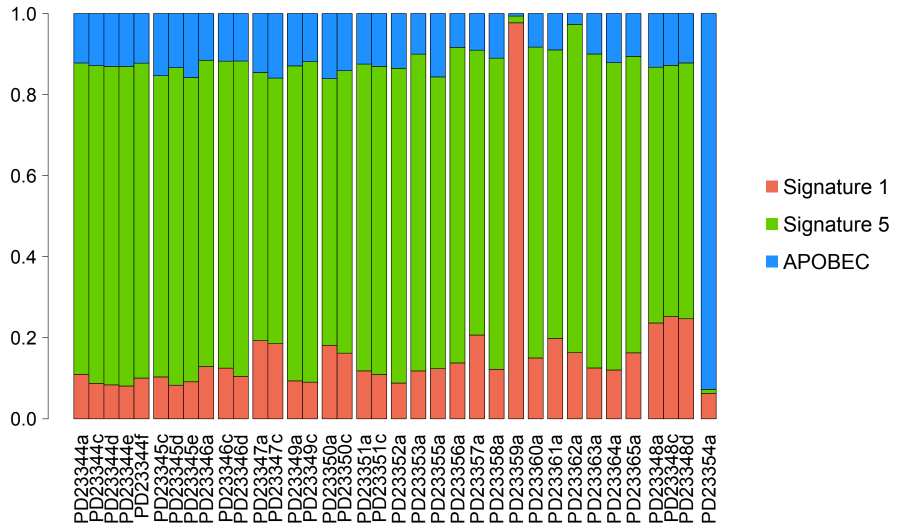

Supplementary S12 | APOBEC mutational signature and *EML4-ALK* fusion identify probably primary lung adenocarconoma metastasis. **(a)** Circos plot of PD23354a. All substitution mutations are represented by their intermutation distance per chromosome and are color coded according to which base substitution (C>A, C>G, C>T, T>A, T>C, T>G). The six concentric circles in each plot, from outermost inwards, represent the human chromosomes, substitutions, insertions, deletions and copy number for each allele. The three bar plots to the right of each circos depict, in descending order, substitution mutation trinucleotide context and burden of small indels and rearrangements. **(b)** Bar plot of the relative contributions of the three predominant signatures identified in the intial tumor cohort. PD23354a (far right) demonstrated strong evidence of a signaure attributed to activity of the AID/APOBEC family of cytidine deaminases, and not present to any significant degree in any of the other samples.

Supplementary Figure S13

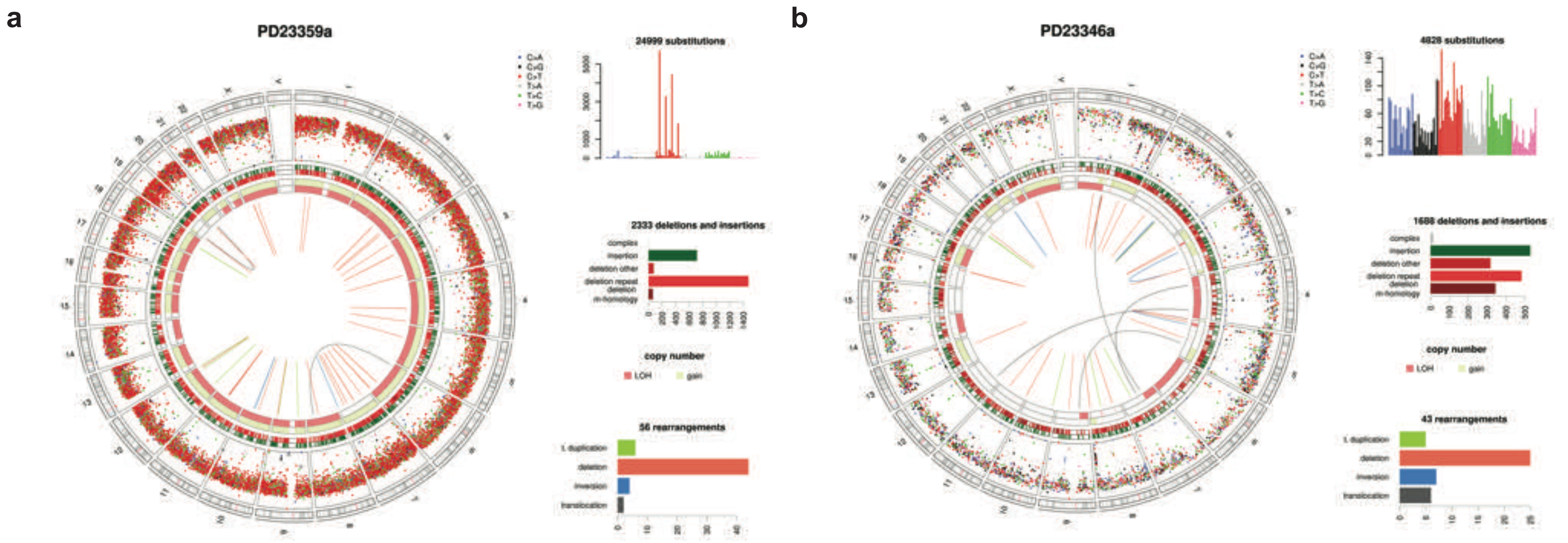

Supplementary Figure S13 | A hypermutator anaplastic meningioma with near complete loss of heterozygosity. (a) Circos plot of PD23359a, a primary anaplastic meningioma with a hypermutator phenotype and extensive loss of heterozygosity. **(b)** Displayed alongside for comparison is the circos plot of a typical anaplastic meningioma (PD23346a). All substitution mutations are represented by their intermutation distance per chromosome and are color coded according to which base substitution (C>A, C>G, C>T, T>A, T>C, T>G). The six concentric circles in each plot, from outermost inwards, represent the human chromosomes, substitutions, insertions, deletions and copy number for each allele. The three bar plots to the right of each circos depict, in descending order, substitution mutation trinucleotide context and burden of small indels and rearrangements.
